# Sensor acquired reachable workspace in the elderly population: A cross-sectional observational study

**DOI:** 10.1097/MD.0000000000029575

**Published:** 2022-07-29

**Authors:** Vicky Chan, Richard Thai, Revik Vartanian, Min Su Kim, Maya N. Hatch, Jason Koh, Jay J. Han

**Affiliations:** a University of California at Irvine School of Medicine, Department of Physical Medicine and Rehabilitation, Irvine, CA, USA; b University College of Medicine and Institute of Wonkwang Medical Science, Department of Rehabilitation Medicine, Iksan, Republic of Korea.

**Keywords:** elderly, kinect, reachable workspace, upper extremity

## Abstract

The elderly population experiences a decline in upper extremity range of motion (ROM), impairing activities of daily living. The primary mode of quantification is by goniometer measurement. In this cross-sectional observation study, we investigate a sensor-acquired reachable workspace for assessing shoulder ROM decline in an elderly population in comparison to traditional measurements.

Sixty-one healthy subjects aged ≥ 65 years were included and compared to a cohort of 39 younger subjects, aged 20 to 64. A sensor acquired reachable workspace using a Kinect motion capture camera measured the maximum reaching ability of both arms while in a seated position, measured in m^2^ and normalized to arm length to calculate a novel score defined as a relative surface area. This score approximates range of motion in the upper extremity. This measurement was compared to goniometer measurements, including active ROM in shoulder flexion and abduction.

Total RSA shows moderate to strong correlation between goniometer in flexion and abduction in the dominant arm (*R* = 0.790 and *R* = 0.650, *P* < .001, respectively) and moderate correlations for the nondominant arm (*R* = 0.622 and *R* = 0.615, *P* < .001). Compared to the younger cohort, the elderly population demonstrated significantly reduced total RSA in the dominant arm (mean_elderly_ = 0.774, SD = 0.09; mean_younger_ = 0.830, SD = 0.07, *P* < .001), with significant reductions in the upper lateral quadrant in both arms (dominant: mean_elderly_ = 0.225, SD = 0.04; mean_younger_ = 0.241, SD = 0.01; *P* < .001; nondominant: mean_elderly_ = 0.213, SD = 0.03; mean_younger_ = 0.228, SD = 0.01; *P* = .004). The test-retest reliability was strong for both dominant and nondominant total RSA (ICC > 0.762).

The reachable workspace demonstrates promise as a simple and quick tool for clinicians to assess detailed and quantitative active shoulder ROM decline in the elderly population.

## 1. Introduction

### 1.1. Background

Upper extremity range of motion (ROM) is critical for activities of daily living (ADL).^[1]^ Adequate motor control and ROM in the upper extremities are necessary for individuals to perform not only basic ADLs (eating, dressing, grooming, toileting), but also to manipulate and interact with the surrounding environment for work and leisure activities. The magnitude of upper extremity impairment in the elderly population can be significant.^[2]^ According to the 2014 US Census report, there are 8.2 million adults who report having a functional limitation, with 12.4 percent of adults having difficulty with at least 1 ADL.^[3]^ In the same report, adults aged 65 years and older were 3 times as likely to have an upper body functional limitation compared to people between 18 and 24 years old.^[3]^

The extent and detailed characterization of upper extremity ROM and functional decline in the elderly population, however, is limited. ROM measurements using goniometry and manual muscle testing provide only a limited glimpse of the extent of the overall potential functional impairment.^[4,5]^ Both evaluation methods require accessibility and availability of clinicians.^[4,5]^ The general elderly population may not be convinced to seek medical help at an early stage of limitation^[6]^ or may have transportation difficulties in consulting clinicians.^[7]^ Written surveys or self-reported assessments of upper extremity function, such as Disabilities of the Arm, Shoulder, and Hand (DASH), and Penn Shoulder Score (PSS), can be performed at home, but do not provide objective physical quantitative information.^[8]^

The shoulder joint and mobility of the upper extremity are complex interactions that allow physical tasks in ADL involving lifting, grasping, pushing, and pulling. With recent advances in technology and software development, sensor systems such as Kinect (Microsoft, Redmond, WA) paired with kinematic modeling can be used in the clinical setting to acquire body-specific movements that can measure one’s physical capabilities. The use of algorithms or computer software programs to analyze motion is well established in engineering fields for the analysis of mechanical systems such as robots, and can serve as a solution to assess overall upper extremity function from sensor-acquired motion data.^[9,10]^ A sensor-based outcome measure, called the upper-extremity reachable workspace, has been previously developed and has already demonstrated excellent reliability, validity, and sensitivity to change in a variety of neurological and musculoskeletal conditions, including muscular dystrophy,^[11]^ stroke,^[12,13]^ and rotator cuff disorders.^[14]^

### 1.2. Objectives

The purpose of this study was to determine the viability of using the reachable workspace measure as an assessment tool to identify and characterize in detail the upper extremity ROM impairment in the elderly population.

## 2. Methods

### 2.1. Study design

This cross-sectional observational study followed the STROBE statement.^[15]^ This study recruited participants aged ≥ 65 years, who were otherwise self-identified as healthy, to quantify upper extremity ROM using a Kinect motion capture device to measure the maximum reaching ability in a seated position.

### 2.2. Setting

Participants were recruited from university-affiliated senior health clinics and senior centers in Orange County in Southern California from April 2018 to October 2019 using a convenience sample. The study consisted of a single visit where medical history, baseline information, anthropometric information, and clinical measures were obtained.

### 2.3. Sample Size determination

The sample size was determined based on the hypothesis that the Kinect-measured reachable workspace is highly correlated with goniometric measurement in the aging population, specifically at a correlation of 0.80 as compared to a null correlation of 0.50. With a sample size of 60 patients (>65 years old), the study will achieve 99% power to test this hypothesis at 5% significance level.

### 2.4. Participants

Sixty-one participants were enrolled. Participants with confirmed diagnosis of severe neurological, musculoskeletal, or neuromuscular conditions were excluded from this study. In addition to data collected for this study, a previous data set of thirty-nine healthy study participants under the age of 65, collected with the same protocol, was used to compare reachable workspace to the elderly participant data.

### 2.5. Ethical review

The study protocol was approved by the University of California, Irvine Institutional Review Board (IRB) IRB number HS# 2017-3801, and written informed consent was obtained before the start of the study procedures.

### 2.6. Bias

To minimize selection bias, consecutive patients meeting the inclusion criteria were recruited from health clinics and senior centers. Measurement errors were minimized by using a single evaluator throughout the study.

### 2.7. Outcome measures

The primary outcome measures were the novel reachable workspace measurement normalized to arm length and defined as relative surface area (RSA) and upper extremity ROM as measured by goniometer.

#### 2.7.1. Goniometric measurements of shoulder motion in flexion and abduction.

Active ROM in both shoulder flexion and abduction was measured with goniometer. All tests were performed by a single examiner throughout the study. Goniometric measurements were performed with the participants in a sitting position.

All subjects were instructed to sit with their back straight in a standard chair with a seat height of 46 cm, without armrests. They were instructed to keep their elbows extended throughout the motion. No spinal extension or lateral trunk bending was allowed. The testing motion was demonstrated before every measurement. The participants then performed the motion in their available range. Verbal and tactile cueing was provided to guide the movement in the proper plane, if needed. The same ROM was then repeated and measured using a universal 12-inch goniometer.

#### 2.7.2. Upper extremity reachable workspace protocol and analysis.

**Figure 1.**
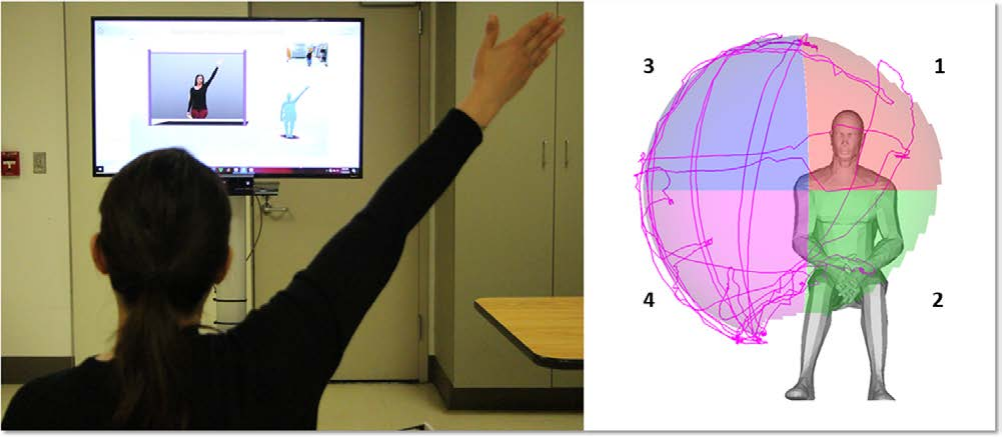
Reachable workspace system set up and relative surface area (RSA) envelope output with 4 quadrants: 1, medial upper quadrant; 2, medial lower quadrant; 3, lateral upper quadrant; 4, lateral lower quadrant (Right shoulder RSA is shown).

**Figure 2.**
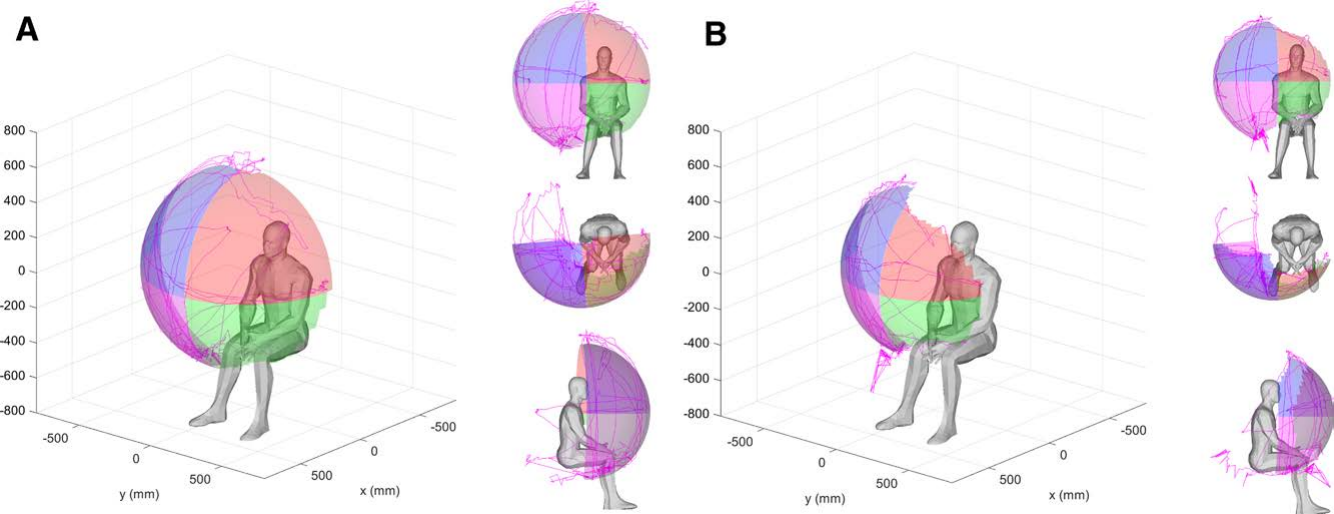
Graphical visualization of 3D relative surface area (RSA) output between 2 different and representative participants A and B. (A) Dominant arm of participant age 36. (B) Dominant arm of participant age 83.

The upper-extremity reachable workspace measurement was performed using the Microsoft Kinect 2.0 sensor (Redmond, WA) following previously published protocols.^[11,13,16,17]^ The subjects sat in front of a motion sensor and moved their arms in a prescribed movement protocol, while the sensor tracked the arm movement, lasting approximately 1.5 minutes. The participants were seated in front of a Microsoft Kinect sensor at a distance of 230 cm. A standard upright chair without armrests, with a seat height of 46 cm, was used. The participants were asked to watch an instructional video that demonstrated the movement protocol prior to performing it. The movements were assessed, and participants were cautioned against the use of compensatory movement patterns. Study participants were then asked to follow the model from the assessment video and perform a set of standardized movements consisting of lifting the arm from the resting position to above the head while keeping the elbow extended, and then performing the same movement in the vertical planes at around 0°, 45°, 90°, and 135°. This set of movements involves shoulder abduction, shoulder scaption, shoulder flexion, horizontal abduction, horizontal adduction, shoulder extension, and shoulder adduction. The second set of movements consisted of horizontal sweeps at the level of the umbilicus and shoulder. Each arm was tested separately. For this study, data collection was performed twice, with a 15-minute rest break between trials. Following the previously published and established protocol,^[11,13,16,17]^ for analysis, the reachable workspace envelope was split into 4 different quadrants (upper medial and lateral, lower medial and lateral), with the shoulder joint serving as the origin. The quadrants were then numbered 1 to 4 relative to each tested arm (1, medial upper quadrant; 2, medial lower quadrant; 3, lateral upper quadrant; 4, lateral lower quadrant). To allow for comparison between patients, absolute total and quadrant reachable workspace surface envelope areas (m^2^) were normalized by each individual arm length to obtain the RSA.^[11,13,16,17]^ The output RSA results are displayed both numerically and visually with spatial mapping, with each quadrant having a maximum value of 0.25 (Figs. 1 and 2).

### 2.8. Statistical analysis

The demographic and clinical characteristics of the study participants are presented as the mean and standard deviation for all continuous variables, and the dichotomous variable, sex, is presented as frequency and percentage. Normality of data was assessed using the Shapiro–Wilk test, and appropriate nonparametric testing was used (Mann-Whitney U and Wilcoxon signed-rank tests). Pearson correlation coefficients were used to determine the relationship between RSA and clinical measures, including goniometry, in the elderly population. The spectra of the RSA and goniometric measured ROM were compared by arm and sex. The Mann–Whitney U test was used to compare the differences in age and sex, and the Wilcoxon signed-rank test was used to compare the arms. For any significant findings (*P* < .05), a planned contrast with adjusted probability values (Benjamini–Hochberg correction) was performed to locate the difference.^[18]^ The test-retest repeatability of the RSA from the reachable workspace was computed using the intraclass correlation coefficient (ICC) with a confidence interval of 95% between test and retest for each quadrant and arm.^[19]^ All statistical analyses were conducted using SPSS version 27 (SPSS Inc., Chicago, IL, USA). For all analyses, a *P* value of < .05, was used as the level of statistical significance.

## 3. Results

### 3.1. Study participants

Table 1 presents baseline information of the participants. 61 elderly participants were enrolled in the study with an average age of 75.20 years old (SD = 6.36), average height of 165.33 cm (SD = 11.09), and average weight of 67.07 kg (SD = 16.13). Slightly less than half of the participants were men (47.5%, n = 29). Data from 39 young healthy controls showed an average age of 38.92 years old (SD = 12.30), an average height of 165.91 cm (SD = 10.54), and an average weight of 70.35 Kg (SD = 14.40). Slightly less than half of the younger controls were men (48.7%, n = 19) Table 1.

**Table 1:** Demographic characteristics of the study participants (N = 61) and young healthy controls (N = 39).

	Elderly population (N = 61)	Young healthy controls (N = 39)
Age, yr (mean ± SD)	75.20 ± 6.36	38.92 ± 12.30
Age range, yr (min, max)	65, 91	20, 64
Sex (n, %)	29 (47.5%) male, 32 (52.5%) female	19 (48.7%) male, 20 (51.3%) female
Height, cm (mean ± SD)	165.33 ± 11.09	165.91 ± 10.54
Weight, kg (mean ± SD)	67.07 ± 16.13	70.35 ± 14.40

No statistically significant differences between the age groups were found for height, weight, or sex (*P* = .564, *P* = .205, and *P* = .909, respectively).

### 3.2. Goniometry

There was no significant difference in goniometer measurements in shoulder flexion and abduction between sexes in the elderly population. When comparing the arms, the dominant side exhibited a greater ROM overall in both flexion and abduction than the nondominant arms (flexion mean difference = 5.115°, SD = 18.32, abduction mean difference = 4.508°, SD = 24.88), with statistically significant differences in flexion (*P* < .001) and abduction (*P* = .001). These significant differences remain significant after Benjamini–Hochberg correction (Table 2).

**Table 2:** Comparison of goniometric measured ROM and relative surface area by arm and sex in the elderly population.

	Flexion (degrees)		Abduction (degrees)			Quadrant 1		Quadrant 2		Quadrant 3		Quadrant 4		Total RSA	
ROM	D	ND	D	ND	Relative Surface Area (RSA)	D	ND	D	ND	D	ND	D	ND	D	ND
Sex					Sex										
Male (mean ± SD) n = 29	159.97 ± 21.79	156.17 ± 11.31	160.79 ± 22.11	158.41 ± 11.71	Male (mean ± SD) n = 29	0.194 ± 0.04	0.218 ± 0.04	0.122 ± 0.03	0.146 ± 0.03	0.220 ± 0.04	0.217 ± 0.02	0.225 ± 0.01	0.219 ± 0.01	0.761 ± 0.09	0.799 ± 0.07
Female (Mean ± SD) n = 32	160.81 ± 18.36	154.50 ± 13.99	156.53 ± 25.77	150.09 ± 22.26	Female (mean ± SD) n = 32	0.196 ± 0.06	0.209 ± 0.05	0.132 ± 0.03	0.156 ± 0.03	0.230 ± 0.03	0.209 ± 0.04	0.227 ± 0.01	0.219 ± 0.01	0.785 ± 0.09	0.793 ± 0.09
*P* value (Mann–Whitney U test)	.931	.756	.885	.134	*P* value (Mann–Whitney U test)	.573	.718	.236	.097	.266	.272	.453	.965	.488	.931
Arm sides					Arm sides										
Dominant - nondominant (mean difference ± SD) N = 61	5.115 ± 18.32		4.508 ± 24.88		Dominant - nondominant (mean difference ± SD) N = 61	–0.019 ± 0.05		–0.024 ± 0.03		0.013 ± 0.04		0.007 ± 0.01		–0.022 ± 0.09	
*P* value (Wilcoxon signed ranks test)	<.001*,*,*		**.001** †		*P* value (Wilcoxon signed ranks test)	**<.001** ‡		**<.001** ‡		**<.001** ‡		**<.001** ‡		.01*	

### 3.3. Reachable workspace in elderly

The RSA for all 4 quadrants was found to be significantly different when comparing the dominant and nondominant arms. Dominant quadrants Q1 and Q2 demonstrated a significantly lower RSA (Q1 mean difference = –0.019, SD = 0.05; Q2 mean difference = –0.024, SD = 0.03) than the nondominant arm (*P* < .001), whereas dominant Q3 (mean difference = 0.013, SD = 0.04) and Q4 (mean difference = 0.007, SD = 0.01) exhibited a greater RSA than the nondominant arm (*P* < .001). The total RSA difference between the arms was found to be significant (mean difference = ––0.022, SD = 0.09) but did not remain significant after Benjamini–Hochberg correction (Table 2).

Females had a higher RSA in all 4 quadrants (Q1 mean = 0.196, SD = 0.06; Q2 mean = 0.132, SD = 0.03; Q3 mean = 0.230, SD = 0.03; Q4 mean = 0.227, SD = 0.01) and total RSA (mean = 0.785, SD = 0.09) in the dominant arm than males, but these differences were not statistically significant (Table 2).

### 3.4. Correlations between reachable workspace and goniometric measurement

There was a strong positive correlation between the RSA of the reachable workspace with goniometric measurements for shoulder flexion and abduction in the dominant arm in Q3 (Pearson correlation coefficient, *R* = 0.878, *R* = 0.786, respectively, *P* < .001), combined Q1 and Q3 (*R* = 0.806, *R* = 0.784, respectively, *P* < .001), and Q3 and Q4 (*R* = 0.881, *R* = 0.773, respectively, *P* < .001). The total RSA of the dominant arm in flexion (*R* = 0.790, *P* < .001) and the nondominant arm in Q3 (*R* = 0.793, *P* < .001) and Q3 and Q4 (*R* = 0.783, *P* < .001) in shoulder abduction also showed a strong correlation. A moderate correlation was found in Q1 for both dominant and nondominant flexion (*R* = 0.579, *R* = 0.568, *P* < .001) and abduction (*R* = 0.607, *R* = 0.486, respectively, *P* < .001). Quadrants 2 and 4 showed the lowest correlation (Table 3).

**Table 3:** Correlation between relative surface area of reachable workspace and goniometric ROM measurement.

	D RSA Q1		D RSA Q2		D RSA Q3		D RSA Q4		D Total RSA		D RSA Q1 and Q2		D RSA Q1 and Q3		D RSA Q3 and Q4	
Goniometric ROM measurement (N = 61)	r	*P* value	r	*P* value	r	*P* value	r	*P* value	r	*P* value	r	*P* value	r	*P* value	r	*P* value
Dominant flexion	0.579	<.001	0.195	.131	0.878	<.001	0.299	.019	0.790	<.001	0.560	<.001	0.806	<.001	0.881	<.001
Dominant abduction	0.607	<.001	0.153	.239	0.786	<.001	0.202	.118	0.650	<.001	0.565	<.001	0.784	<.001	0.773	<.001
	***ND RSA Q1***		**ND RSA Q2**		**ND RSA Q3**		**ND RSA Q4**		**ND Total RSA**		**ND RSA Q1 and Q2**		**ND RSA Q1 and Q3**		**ND RSA Q3 and Q4**	
	**r**	**P value**	**r**	**P value**	**r**	**P value**	**r**	**P value**	**r**	**P value**	**r**	**P value**	**r**	**P value**	**r**	**P value**
Nondominant Flexion	0.568	<.001	0.096	.460	0.620	<.001	0.244	.058	0.622	<.001	0.508	<.001	0.668	<.001	0.601	<.001
Nondominant abduction	0.486	<.001	0.000	.999	0.793	<.001	0.362	.004	0.615	<.001	0.396	.002	0.684	<.001	0.783	<.001

### 3.5. Reachable workspace compared to younger population

**Figure 3.**
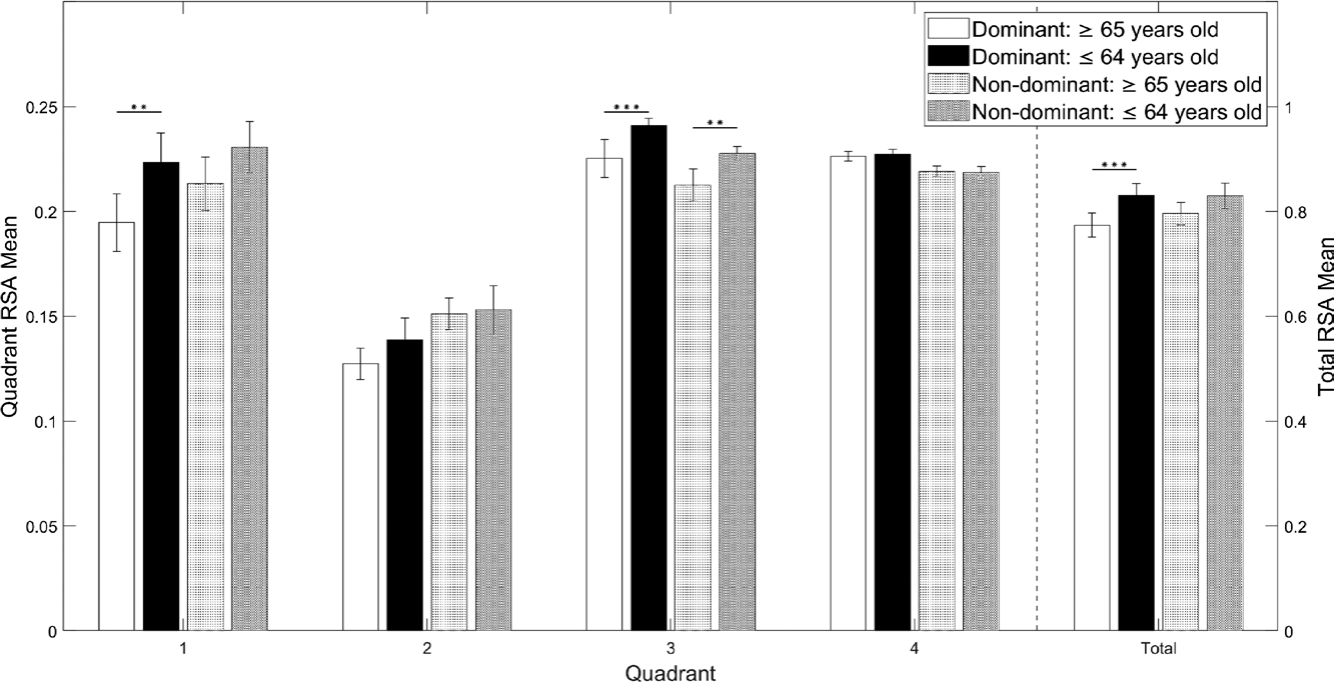
Bar graph of the mean relative surface area (RSA) by age, arm dominance, individual and total quadrants. Bar graph showing the gradual decrease in RSA as age increases. The asterisk (*) means the significant differences between the elderly group and the younger control group in upper extremity function (**P < .01, ***P < .001). Error bars were set at 95% of confidence interval.

Total RSA declined in both dominant and nondominant arms with increasing age, with the decline in dominant arm reaching significance when comparing the elderly population to the younger population (mean_elderly_ = 0.774, SD = 0.09; mean_younger_ = 0.830, SD = 0.07, *P* < .001) (Table 4, Figs. 2 and 3).

**Table 4:** Comparison of relative surface area from reachable workspace between the elderly population and younger controls.

Relative surface area (RSA) - dominant arms	Quadrant 1	Quadrant 2	Quadrant 3	Quadrant 4	Total RSA
Elderly population (mean ± SD) n = 61	0.195 ± 0.05	0.127 ± 0.03	0.225 ± 0.04	0.226 ± 0.01	0.774 ± 0.09
Younger controls (mean ± SD) n = 39	0.223 ± 0.04	0.139 ± 0.03	0.241 ± 0.01	0.227 ± 0.01	0.830 ± 0.07
*P* value (Mann–Whitney U test)	**.003** *	.055	**<.001** †	.963	**<.001** †
**Relative surface area (RSA)–nondominant arms**	**Quadrant 1**	**Quadrant 2**	**Quadrant 3**	**Quadrant 4**	**Total RSA**
Elderly population (mean ± SD) n = 61	0.213 ± 0.05	0.151 ± 0.03	0.213 ± 0.03	0.219 ± 0.01	0.796 ± 0.08
Younger controls (mean ± SD) n = 39	0.231 ± 0.04	0.153 ± 0.04	0.228 ± 0.01	0.219 ± 0.01	0.830 ± 0.07
*P* value (Mann-Whitney U test)	.051	.941	**.004** *	.700	.148

When examining individual quadrants, the RSA of the older population showed a significant decline for both dominant arms in Q3 (mean_elderly_ = 0.225, SD = 0.04; mean_younger_ = 0.241, SD = 0.01; *P* < .001) and nondominant arms in Q3 (mean_elderly_ = 0.213, SD = 0.03; mean_younger_ = 0.228, SD = 0.01; *P* = .004). There was a significant difference in Q1 in the dominant arm (mean_elderly_ = 0.195, SD = 0.05; mean_younger_ = 0.223, SD = 0.04; *P* = .003) when the nondominant arm approached significance (mean_elderly_ = 0.213, SD = 0.05; mean_younger_ = 0.231, SD = 0.04; *P* = .051). All significance remained after the Benjamini–Hochberg correction (Table 4, Figs. 2 and 3).

### 3.6. Test retest reliability of reachable workspace

For RSA measurement in the elderly population, the overall total RSA test-retest reliability was good for both the individual dominant and nondominant arms (intraclass correlation coefficient ICC = 0.762 and ICC = 0.776, respectively). Quadrant 3 showed the highest reliability among all quadrants for both the dominant and nondominant arms (ICC = 0.891 and ICC = 0.817). Quadrants 2 and 4 had moderate test-retest reliability for the dominant side (ICC = 0.481 and ICC = 0.580). Quadrant 2 had moderate reliability for the nondominant side (ICC = 0.511) (Table 5).

**Table 5:** Test retest reliability with intraclass correlation coefficient.

Arm Side	RSA	N	ICC (95% CI; *P* value)
Dominant	Quadrant 1	61	0.607 (0.422, 0.744; *P* < .001)
	Quadrant 2	61	0.481 (0.264, 0.652; *P* < .001)
	Quadrant 3	61	0.891 (0.826, 0.933; *P* < .001)
	Quadrant 4	61	0.580 (0.387, 0.725; *P* < .001)
	Total RSA	61	0.762 (0.634, 0.850; *P* < .001)
Nondominant	Quadrant 1	61	0.759 (0.629, 0.848; *P* < .001)
	Quadrant 2	61	0.511 (0.300, 0.674; *P* < .001)
	Quadrant 3	61	0.817 (0.714, 0.886; *P* < .001)
	Quadrant 4	61	0.753 (0.621, 0.844; *P* < .001)
	Total RSA	61	0.776 (0.653, 0.859; *P* < .001)

## 4. Discussion

In this cross-sectional observational study of upper extremity ROM, the elderly group demonstrated a smaller RSA in all 4 quadrants compared to the younger population, indicating a decreased overall ROM. Among the elderly population, major differences were seen in Q1 and Q2 between the dominant and nondominant arms, with the dominant side showing a decrease in RSA vs the nondominant side. The opposite was found for Q3 and Q4, with the dominant arm exhibiting greater RSA. Differences in RSA between males and females were also found, with the greatest difference seen in Q2. Most importantly, RSA showed a moderate to strong correlation with active shoulder ROM, as measured by a standard goniometer. Results of this study demonstrate that measuring ROM using a Kinect sensor is feasible, reliable, and comparable to goniometer ROM measurements in the elderly population.

Data from this study shows that the reachable workspace outcome measure can detect decreased reachability in all 4 quadrants in the elderly population as measured by RSA, when compared to younger controls. These findings are consistent with the existing literature that show predictable decline in shoulder ROM in the elderly population measured by goniometer, as previously reported by Desrosiers,^[20]^ Fiebert,^[21]^ and by inclinometer, as reported by Gill.^[22]^ Overall, the decline in region-specific areas of the RSA in the elderly population is most pronounced in the upper regions of the reaching sphere, Q1, Q3, measuring shoulder flexion and abduction. This difference is significant in the dominant arm and approaches significance in the nondominant arm in the elderly group. This limited ability to fully extend and internally or externally rotate the shoulder may be related to rotator cuff tendinitis or rupture, bursitis, subacromial impingement, acromioclavicular joint osteoarthritis, frozen shoulder, or proximal humerus fractures due to osteoporosis.^[6]^ Decreased ROM in the elderly population may be due to an age-related decline in full shoulder flexion and abduction,^[20]^ which could be the result of poor posture and prior activity level or occupation affecting shoulder flexion and abduction vs mechanical limitations, such as muscle, ligament, or tendon injury.^[21,23]^

Hand dominance in the elderly shows significant effect in reachable workspace. When the dominant arm reached across the body, the RSA was significantly lower than when the nondominant arm reached across the body. It is not known whether differences in hand dominance plays a role in ROM or if the difference is attributed to tension in the shoulder capsule or ligament from increased use in the dominant arm that is not seen as compared to the nondominant side. Murray^[24]^ observed no difference in ROM in goniometry between dominant and nondominant sides when observing younger and older groups. While the limitation of reaching across the body of the dominant arm, Q1 and Q2, is seen in this study, the opposite is true for reaching away from the body in Q3 and Q4. There was a greater RSA in the dominant arm as compared to the nondominant arm. This finding is confirmed by goniometer measurements showing greater shoulder flexion and abduction in the dominant arm and correlates with Barnes et al^[25]^ and Gill et al,^[22]^ that nondominant arms have greater internal rotation in ROM, while dominant arms have greater external rotation.^[25]^

Differences in reachable workspace between elderly males and females were also found in this study. This finding was previously demonstrated by Han^[16]^ as significant in Q2 using the reachable workspace measure while Clement^[26]^observed significant differences in Q1 and Q4 using the same measure. This study demonstrated differences in both Q1 and Q4 of the dominant arm and Q2 in both arms, but none reached significance. These differences in shoulder ROM have been inconsistent in literature but may be associated with females maintaining a greater ROM, especially rotational movement of the shoulder compared with males.^[27]^ This was attributed to several factors, such as greater flexibility in females^[20,21]^ and structural anatomical differences. Males typically have more pronounced muscle development in upper limb internal rotators, limiting their rotational ROM.^[27]^ However, another study by Gibson^[28]^ using the Western Ontario Rotator Cuff Index, a disease-specific quality of life questionnaire designed for rotator cuff disorder patients, suggested that older women demonstrated poorer upper extremity function than older men, assuming that lifestyle differences between men and women could be the main cause. Some researchers have also suggested that there is no difference in reaching capability or upper extremity function between sex.^[26]^

The strengths of this study include a large sample size with all study participants able to follow the simple guided motion protocol while sitting in front of the sensor to acquire the RSA. This study had several limitations. The goniometric measurements of shoulder ROM were performed in the upright position for flexion and abduction only. The recommended position for testing shoulder flexion and abduction using a goniometer is supine.^[28]^ In this study, all participants were tested in a sitting position to be consistent with the testing position for the upper-extremity reachable workspace. Sabari et al,^[29,30]^ indicates that the intrarater reliability for active measurement of shoulder flexion and abduction in the sitting position is extremely high (ICC = 0.97, 0.97, respectively) as long as testing is administered in a consistent position. Each goniometric measurement was performed in a single plane of motion to align with the anatomical structure. The reachable workspace, however, is a measurement incorporating rotational movements that would be closer to functional movement, such as reaching. Direct comparison of RSA as an adjunct to goniometric measurements may only provide an approximation of the true value.

Physical impairments, decline, and limitations occur at different rates as people age. The elderly population may suffer from upper extremity limitations, which may affect their ability to perform ADL. This study confirms a decline in upper extremity ROM in the elderly as compared to a younger cohort. This decline is likely age related and is most pronounced in the upper reaching sphere. Hand dominance and gender may play a role in overall ROM decline. The elderly may not seek medical advice because of their limitations.^[6]^ Knowledge of these limitations and assessment of the need for treatment can improve clinical outcomes and quality of life. This study shows the viability of a quantitative measurement of upper extremity ROM in an elderly population as measured by a motion capture sensor to calculate RSA. Recommendations for future investigations may involve comparing a population with known limitations of ADL’s and measuring reachable workspace to determine a minimum RSA needed to function.

## 5. Conclusions

The reachable workspace outcome is a viable assessment that can be performed quickly to accurately measure upper extremity ROM in the elderly population. Early identification of limitations in ROM that may affect ADL and providing therapy can improve quality of life.

## Acknowledgements

The University of California at Irvine Biostatistics, Epidemiology, and Research Design Department was used in consultation for statistical analysis.

## Author contributions

J.H., J.K., R.V., and V.C. were involved in the concept and design of the study. R.T. and V.C. collected and analyzed subject data. V.C., M.K., M.H., and J.H. were involved in drafting the manuscript. All authors reviewed and approved the final version of the manuscript.

## Footnotes

* Statistically significant at *P* < .05.

† Statistically significant at *P* < .01.

‡ Statistically significant at *P* < .001.

* Statistically significant at *P* < .01.

† Statistically significant at *P* < .001.
